# Effect of NLRP3 inflammasome induced astrocyte phenotype alteration in morphine tolerance

**DOI:** 10.3389/fphar.2024.1434295

**Published:** 2024-11-12

**Authors:** Zhenyu Yuan, Boxuan Lu, Meiling Zhang, Yinxiao Lu, Zhihui Wang, Wenhao Zhang, Hao Cheng, Zhifang Wu, Qing Ji

**Affiliations:** Department of Anesthesiology, Nanjing Jinling Hospital, Affiliated Hospital of Medical School, Nanjing University, Nanjing, China

**Keywords:** NLRP3 inflammasome, morphine tolerance, reactive astrocytes, MCC950, astrocyte phenotype, neuroinflammation

## Abstract

**Introduction:**

Morphine is a widely used analgesic, but its prolonged use often leads to tolerance, limiting its therapeutic efficacy. Research implicates the NLRP3 inflammasome and reactive astrocytes in the development of morphine tolerance, with reactive astrocytes classified into A1 neurotoxic and A2 neuroprotective phenotypes. This study explores the role of the NLRP3 inflammasome and the transformation of astrocyte phenotypes in the progression of morphine tolerance.

**Methods:**

A model of morphine tolerance was established by administering morphine intrathecally for seven consecutive days. To inhibit NLRP3 inflammasome activation, we coadministered MCC950, a selective NLRP3 inhibitor. Thermal withdrawal latency was used to assess tolerance development. Protein and mRNA levels of GFAP, IL-18, NLRP3, C3 (A1 marker), and S100A10 (A2 marker) in the spinal cord were measured using Western blotting (WB) and real-time quantitative polymerase chain reaction (RT-qPCR). Immunofluorescence was employed to assess the colocalization of C3 and GFAP.

**Results:**

Seven days of morphine administration induced tolerance, which was associated with increased levels of GFAP, IL-18, NLRP3, and C3, and a decreased level of S100A10. Coadministration of morphine and MCC950 significantly slowed the development of morphine tolerance and reversed changes in NLRP3, IL-18, GFAP, C3, and S100A10 protein levels.

**Discussion:**

Our findings indicate a significant link between NLRP3 inflammasome activation and morphine tolerance, suggesting that NLRP3 contributes to the transformation of astrocytes to the A1 phenotype. Inhibiting NLRP3 inflammasome activation holds promise in reversing astrocyte phenotype changes, potentially mitigating morphine tolerance.

## Introduction

Morphine is extensively employed as a pain-relieving medication in both immediate and prolonged pain management within clinical settings. However, its clinical application is hindered by notable side effects, including drug tolerance and dependence. The mechanisms of morphine tolerance include desensitization of the μ-opioid receptor (MOR) (Williams et al., 2013), upregulation of pro-inflammatory cytokines (Liu et al., 2019), formation of mu/delta heterodimers (Pasternak and Pan, 2011), activation of the adenosine 3′,5′-cyclic monophosphate (cAMP) pathway (Nestler and Aghajanian, 1997), activation of the p38-MAPK pathway (Fu and Zhang, 2023) and others. Recent studies have demonstrated that glial cells play an important role in the development of morphine tolerance.

Astrocytes, which constitute more than fifty percent of the brain’s cellular population, perform essential physiological functions (Molofsky and Deneen, 2015) and are implicated in the pathophysiological processes of various central nervous system diseases (Blanco-Suárez et al., 2017). Astrocytes regulate neuronal function through cytokine secretion, and specific chemokines and cytokines contribute to morphine tolerance. Notably, interleukin-1β (IL-1β), secreted by astrocytes, is thought to play a crucial role in the development of morphine tolerance. Previous studies reported that co-injection of morphine and IL-1 receptor antagonist (IL-1Ra) could delay the development of morphine tolerance. Furthermore, the chemokine CX3CL1 is also reported to be involved in the activation of astrocyte and the development of morphine tolerance. Inhibiting CXCL1 receptor CXCR1 has demonstrated the capacity to delay morphine tolerance (Johnston et al., 2004) and mitigate astrocyte activation (Cho et al., 2021). These discoveries strongly support the significant involvement of neuroinflammation and astrocyte activation in the progression of morphine tolerance.

It is worth noting that the proliferation of reactive astrocytes exhibits significant heterogeneity. In the context of cerebral ischemia, these astrocytes release neurotrophic factors and cytokines (Zamanian et al., 2012), which help repair the blood-brain barrier, limit immune cell influx, and reduce neuronal death (Sofroniew, 2009). Conversely, reactive astrocytes induced by LPS activate the classical complement pathway, participating in synaptic regulation and theorized to be involved in the progression of neurodegenerative disorders (Zamanian et al., 2012). Reactive astrocytes can be classified as A1 astrocytes (neurotoxic) or A2 astrocytes (neuroprotective) (Liddelow et al., 2017). Furthermore, growing evidence suggests that A1 phenotype produced by reactive astrocytes is detectably expressed in various CNS disorders, including Alzheimer’s disease (AD), Parkinson’s disease (PD), Amyotrophic lateral sclerosis (ALS) (Goetzl et al., 2018; Yun et al., 2018; Kwon and Koh, 2020) and spinal cord injury (Qian et al., 2019; Zhang et al., 2021). Nevertheless, the role of A1 reactive astrocytes in morphine tolerance remains unclear.

The nucleotide-binding oligomerization domain-like receptor pyrin domain-containing-3 (NLRP3) inflammasome is a protein complex that comprises a cytosolic sensor, an adaptor protein (apoptosis-associated speck-like protein containing a caspase-recruitment domain, ASC) and an effector, procaspase-1. It serves as a central component in the host’s immune reactions to infections or tissue injury (O’Brien et al., 2020). Upon detecting pathogens or stress signals, NLRP3 binds to ASC and procaspase-1, forming the NLRP3 inflammasome and resulting in subsequent reactions downstream, such as the maturation and release of interleukin-1β (IL-1β) and IL-18 (Schroder and Tschopp, 2010; Zhong et al., 2013; Shao et al., 2015).

Inflammasome dysregulation is implicated in neurological diseases such as multiple sclerosis (MS), AD, Huntington’s disease (HD), and ALS (Shao et al., 2015). Moreover, the NLRP3 inflammasome is also implicated in opioid tolerance. The administration of morphine leads to an increase in TLR4, TAK1, and NLRP3 expression, while mice with a knockout of NLRP3 show resistance to morphine tolerance (Wang et al., 2021). Notably, morphine can also extend chronic neuropathic pain by activating NLRP3 (Grace et al., 2016). Early morphine administration in a spinal cord injury model upregulates NLRP3 and IL-1β gene levels, exacerbating neuropathic pain (Ellis et al., 2016). This indicates that the NLRP3 inflammasome could have a significant impact on pain transmission during opioid tolerance. Meanwhile, mounting evidence indicates that the activation of NLRP3 inflammasome affects astrocyte phenotype (She et al., 2022). There is accumulating evidence suggesting that IL-18 can induce the transformation of astrocytes into the A1 phenotype through the NF-κB pathway (Hou et al., 2020). Additionally, Dimethyl itaconate (DI) has been shown to regulate NLRP3, thereby reducing the production of A1 astrocytes stimulated by LPS (Darvish et al., 2022). These studies collectively suggest that the NLRP3 inflammasome may serve as a pivotal factor in governing the differentiation between A1 and A2 astrocytes. However, it remains unclear whether NLRP3 inflammasome actively participate in the phenotypic transformation of reactive A1 astrocytes, thereby contributing to the neurotoxic response in the context of morphine tolerance.

In this study, we observed a notable rise in the expression of NLRP3 and C3, an indicator of A1 reactive astrocytes, in the spinal cord of morphine-tolerant rats. Additionally, the concurrent administration of morphine alongside the specific NLRP3 inhibitor MCC950 not only alleviated the onset of morphine tolerance but also hindered the conversion of reactive astrocytes into the A1 phenotype.

## Experimental procedures

### Animals

Male Sprague Dawley rats, aged 6 weeks and weighing between 200 and 220 g, were sourced from the Animal Center of Jinling Hospital, Nanjing, China. They were housed in cages with 4-5 rats per group, under a 12-h light/dark cycle, in a temperature-controlled environment maintained at 24°C ± 1°C. Rats had *ad libitum* access to food and water. All experimental procedures were conducted in accordance with the Guidelines for the Care and Use of Laboratory Animals provided by the National Institutes of Health and were approved by the Ethics Committee of Jinling Hospital.

### Placement and verification of intrathecal catheter

The method of intrathecal catheter placement was described by previous studies (Cheng et al., 2020). Briefly, under isoflurane anesthesia, we inserted an intrathecal catheter (PE-10, AnLai Software Technology Co., Ltd., Ningbo, China) through the L5 and L6 intervertebral space, extending it to the subarachnoid space of the lumbar enlargement. We secured the internal part of the catheter within the paravertebral muscles, and the outer part onto the skin to prevent accidental dislodgement. The catheter was filled with 15 µL normal saline and the tip of catheter was closed by cauterization. Following the implantation of the intrathecal catheter, each rat was housed individually for a period of 3 days to allow for recovery before proceeding with additional testing. To confirm proper implantation, a 15 µL injection of 2% lidocaine was administered through the intrathecal catheter. Rats demonstrating complete paralysis of the tail and bilateral hind legs, while maintaining normal baseline neurological functions, were exclusively chosen for subsequent experiments. This procedure ensures the accurate placement of the catheter.

### Drug administration

MCC950 (No. B7946), a highly selective inhibitor of NLRP3 inflammasome, was purchased from APExBIO. Rats were partitioned into four groups, with each group receiving either normal saline, morphine (diluted to 15 µg/10 µL with normal saline), or MCC950 (diluted to 2 µg/µL with normal saline) via intrathecal injection for seven consecutive days (D1–D7): NS + vehicle (17.5 µL of saline), NS + MCC950 (10 µL saline +7.5 µL MCC950), morphine + vehicle (10 µL morphine +7.5 µL saline), and morphine + MCC950 (10 µL morphine +7.5 µL MCC950). After each injection, a flush of 15 µL saline was administered through the catheter to ensure the complete delivery of drugs into the subarachnoid space.

### Nociceptive test

To evaluate nociceptive responses, rats were placed in a plastic chamber (22 cm × 12 cm × 22 cm) with a glass floor measuring 3 mm in thickness. Prior to testing, rats were given 15–30 min to acclimate to their surroundings until they stopped exhibiting exploratory behavior. A Plantar Analgesia Meter (IITC Inc., Woodland Hills, CA) served as the radiant heat source, positioned beneath the glass floor and directed towards the rats’ hind paw. Thermal withdrawal latencies (TWLs) were evaluated following the methodology outlined by Hargreaves et al. (1988), wherein TWLs represent the duration from activating the radiant heat source to the occurrence of pain-related behaviors such as paw licking or withdrawal. An automatic 20 s cutoff was set to prevent tissue damage. The thermal pain threshold of the rats was measured before the first day of drug administration and for seven consecutive days after drug administration each day. The results obtained prior to the first day of drug administration were recorded as the baseline thermal pain threshold (BL). Each rat was measured five times with 5-min intervals between measurements. After excluding the maximum and minimum values, the mean thermal latency was calculated and used for further analysis. Data were expressed in two forms: (1) absolute thermal withdrawal latency (TWL) and (2) percentage of maximal possible analgesic effect (%MPE) which was calculated using the formula $\%\text{MPE}=\left[\left(\text{TWL}-\text{BL}\right)\;/\;\left(20-\text{BL}\right)\;\right]\times 100$ , as described previously (Mao et al., 1994).

### Western blot

Rats were deeply anesthetized with isoflurane and underwent transcardial perfusion with 250 mL of ice-cold phosphate-buffered saline. Following perfusion, the fifth lumbar segments of the spinal cord were extracted and homogenized using ice-cold RIPA lysis buffer (Beyotime, P0013B) containing a proteinase and phosphatase inhibitor cocktail (Beyotime, P1045). Following a 30-min incubation on ice and centrifugation at 12,000 rpm for 5 min at 4°C, the supernatants were collected. Protein content was assessed using a BCA protein quantitation kit (Beyotime, P0012). Subsequently, the total protein sample was denatured by boiling in SDS-PAGE Sample Loading Buffer (Beyotime, P0015) for 10 min. Equal quantities of total protein were loaded onto 10% SDS-polyacrylamide gels and subsequently transferred to polyvinylidene fluoride membranes (Millipore, Billerica, MA, United States). The membranes were then incubated in 5% non-fat milk containing 0.02% Tween (TBST) for 1 h at room temperature to block nonspecific binding sites. The membranes were then incubated overnight at 4°C with the following primary antibodies: Goat anti-C3d (1:2000; R&D Systems, United States, AF2655, RRID:AB_2066622), Rabbit anti-NLRP3 (1:2000; abcam, United States, ab263899, RRID:AB_2889890), Rabbit anti-S100A10 (1:2000; Proteintech, China, 11250-1-AP, RRID:AB_2269906), Rabbit anti-IL-18 (1:2000; Proteintech, China, 10663-1-AP, RRID:AB_2123636), Mouse anti-GFAP (1:1,000; Cell Signaling Technology, United States, Cat# 3670, RRID:AB_561049) and Mouse anti-Beta Actin (1:5000; Proteintech, China, 81115-1-RR, RRID:AB_2923704). Following overnight incubation, the membrane was subjected to three washes with TBS containing Tween (TBST), with each wash lasting 5 min. Then add the corresponding horseradish peroxidase (HRP)-conjugated secondary antibody and incubate on a shaker at room temperature for 1 h. After the incubation period, the membrane underwent three washes with TBST, each lasting 5 min. Protein detection was conducted using an enhanced chemiluminescence (ECL) kit from Millipore (Billerica, MA, United States), and the intensities of specific protein bands were quantified using ImageJ software (developed by Wayne Rasband at NIH, MD, United States).

### Real-time quantitative polymerase chain reaction (RT-qPCR)

Total RNA was isolated from spinal cord tissue using Trizol reagent (Invitrogen, Carlsbad, CA, United States), followed by reverse transcription into cDNA using the RevertAid First Strand cDNA Synthesis Kit (K1622; Thermo Fisher Scientific, Waltham, MA, United States). RT-qPCR analysis was conducted using SYBR Green Master Mix (Roche) in accordance with the manufacturer’s protocols. The primer sequences were as follows: NLRP3: F: 5′-GAG​CTG​GAC​CTC​AGT​GAC​AAT​GC-3′, R: 5′-GAG​CTG​GAC​CTC​AGT​GAC​AAT​GC-3′; IL-18: F:5′-ATATCGACCGAACAGCCAAC-3′, R:5′-TTCCATCCTTCACAGATAGGG-3′; S100A10: F:5′-CCTCTGGCTGTGGACAAAAT-3′, R:5′-CTGCTCACAAGAAGCAGTGG-3′; C3: F:5′-AAAAGGGGCGCAACAAGTTC-3′, R:5′-GATGCCTTCCGGGTTCTCAA-3′; GAPDH: F:5′-GGCCTTCCGTGTTCCTACC-3′, R:5′-CGCCTGCTTCACCACCTTC-3′. The NLRP3, IL-18, C3, S100A10 expression was normalized to GAPDH gene expression, and relative quantification was determined utilizing the comparative CT method (2^−ΔΔCT^).

### Immunohistochemistry

After being deeply anesthetized with isoflurane, rats underwent transcardial perfusion with 250 mL of ice-cold PBS buffer, followed by 250 mL of a 4% paraformaldehyde (4% PFA) solution. After perfusion, the rat spinal cord was dissected and immersed in fixative for 24 h, then the tissue was removed, trimmed, and placed in an embedding frame for dehydration and paraffin embedding. The trimmed paraffin block was placed into a microtome for slicing, with a thickness of 4 µm. After dewaxing and antigen retrieval, the slides were blocked with BSA for 30 min, then incubated overnight at 4°C for the following antibody: Goat anti-C3d (1:2000; R&D Systems, United States, AF2655, RRID:AB_2066622) and Mouse anti-GFAP (1:1000; Cell Signaling Technology, United States, Cat# 3670, RRID:AB_561049). Following three washes (5 min each), the slides were incubated with the appropriate secondary antibody at room temperature in the dark for 50 min. Subsequently, they were stained with DAPI staining solution at room temperature in the dark for 10 min. After each incubation, the slides were washed three times with PBS for 5 min each time. Then the slides were treated with the autofluorescence quencher, and washed with distilled water for 10 min. They were then mounted with anti-fade mounting medium and observed under a fluorescence microscope.

### Statistical analysis

The experimental data were analyzed and graphed using GraphPad Prism 9 (http://www.graphpad.com/, RRID:SCR_002798). The measurement data were presented as mean ± standard error of the mean (SEM). Two-group comparisons were conducted using independent samples t-tests, while comparisons involving three or more groups were analyzed using two-way analysis of variance (ANOVA). Multiple comparisons were performed using Tukey or Sidak’s test. A significance level of *p* < 0.05 was applied to determine statistical significance.

## Results

### 1. MCC950 administration attenuated the development of morphine tolerance induced by 7 days of intrathecal morphine injection

**FIGURE 1:**
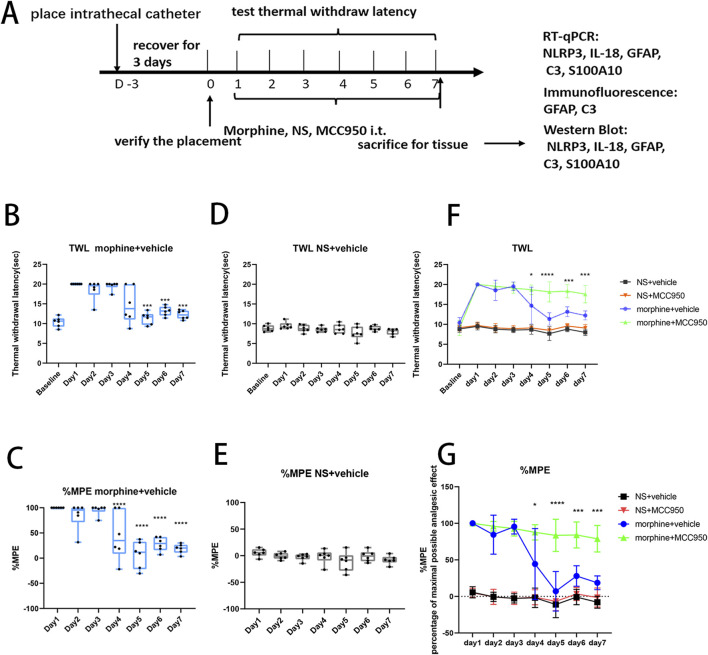
The administration of MCC950 attenuated the development of morphine tolerance induced by 7 days of intrathecal morphine injection. **(A)** Flowchart of the experimental procedure. After the placement of intrathecal catheter, rats received 7 days of intrathecal morphine injection, and the thermal withdrawal latency (TWL) was measured 30 min after each injection. **(B)** The TWL of rats in morphine + vehicle group, compared with day 1, on day 5–7, ***p < 0.001, *p* < 0.05, n = 6 per group. **(C)** The %MPE of rats in morphine + vehicle group, compared with day 1, on day 4–7, ****p < 0.0001, n = 6 per group. **(D)** The TWL of rats in NS + vehicle group, n = 6 per group. **(E)** The %MPE of rats in NS + vehicle group, n = 6 per group. **(F, G)** The TWL and %MPE of rats in the four treatment groups, morphine + vehicle group vs. morphine + MCC950 group, on day 4, *p < 0.05, on day 5, ****p < 0.0001, on day 6–7, ^***^ *p* < 0.001, n = 6 per group.

The analgesic effect of morphine and the development of morphine tolerance was assessed by measuring the TWLs (Figure 1A). After seven consecutive days of morphine administration (15µg, i. t.), the antinociceptive effect of morphine decreased, indicating the development of antinociceptive tolerance to morphine (Figure 1B, two-way ANOVA with Tukey’s multiple comparisons test, day 5, 6, 7 morphine + vehicle vs. day 1 morphine + vehicle; *p* < 0.001, n = 6 per group; Figure 1C, two-way ANOVA with Tukey’s multiple comparisons test, day 4, 5, 6, 7 morphine + vehicle vs. day 1 morphine + vehicle, *p* < 0.0001, n = 6 per group), while no statistical change were observed after repeated saline injection (Figure 1D, two-way ANOVA with Tukey’s multiple comparisons test, day 7 NS + vehicle vs. day 1 NS + vehicle, *p* = 0.3082, n = 6 per group; Figure 1E, two-way ANOVA with Tukey’s multiple comparisons test, day 7 NS + vehicle vs. day 1 NS + vehicle, *p* = 0.8444, n = 6 per group). Meanwhile, compared with morphine + vehicle group, the analgesic effect of morphine was not obviously decreased after 7-days co-administration of morphine and MCC950, a specific NLRP3 inhibitor (Figures 1F, G, two-way ANOVA with Tukey’s multiple comparisons test, day 4 morphine + vehicle vs. day 4 morphine + MCC950, *p* < 0.05, n = 6 per group; day 5 morphine + vehicle vs. day 5 morphine + MCC950, *p* < 0.0001, n = 6 per group; day 6, 7 morphine + vehicle vs. day 6, 7 morphine + MCC950, *p* < 0.001, n = 6 per group).

### 2. MCC950 suppressed the activation of NLRP3 inflammasome and reversed the A1/A2 ratio of reactive astrocytes in morphine tolerance rats

**FIGURE 2:**
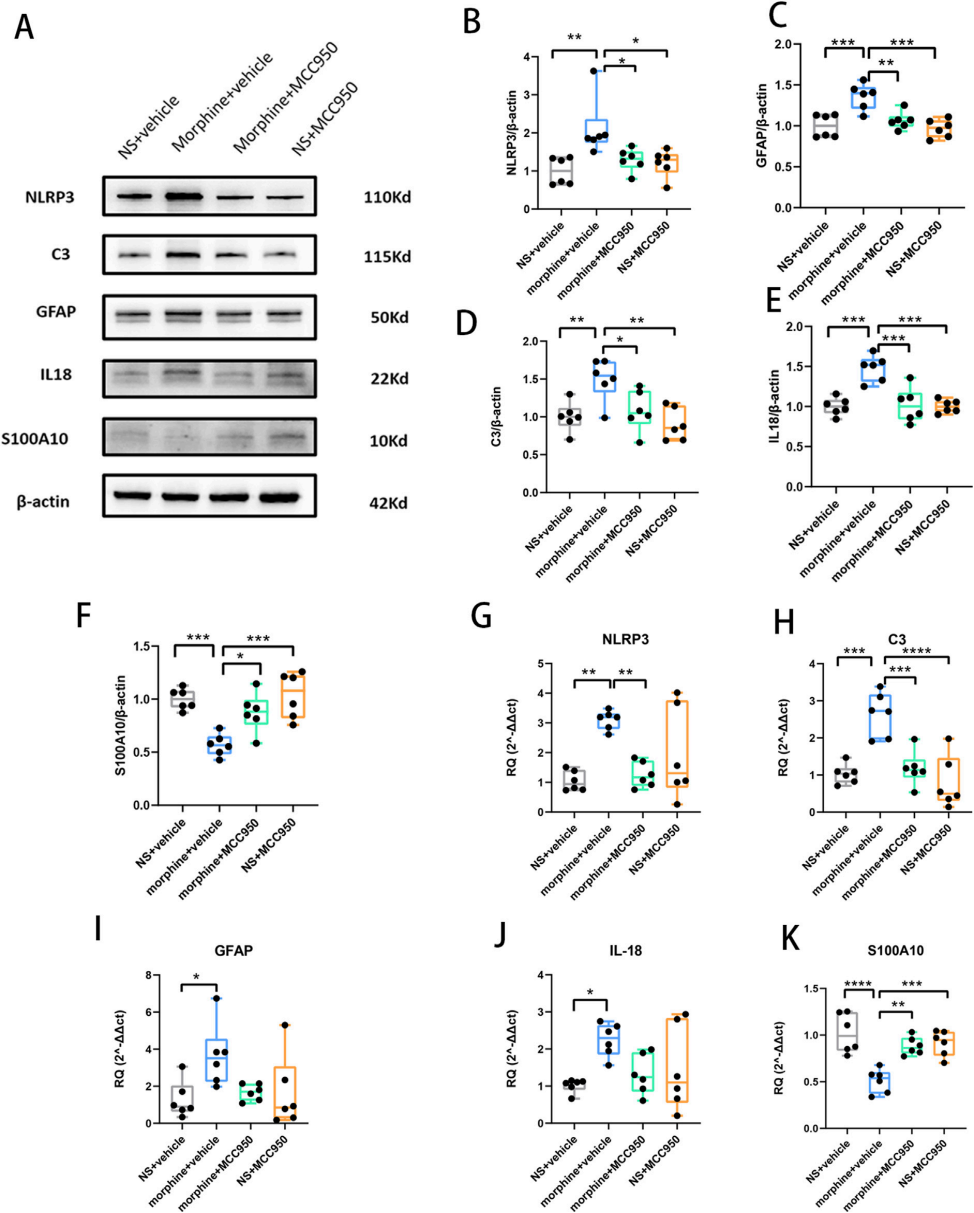
Repeated morphine treatment increased the mRNA and protein levels of NLRP3, C3, GFAP, IL-18, and decreased the expression level of S100A10, while MCC950 alleviated this alteration. **(A)** Representative protein bands in Western blot. **(B–F)** The protein levels of NLRP3, GFAP, C3, IL-18 and S100A10. Graphical data are presented as the mean ± S.E.M and significance was determined by one-way ANOVA followed by Tukey’s test. *p < 0.05, **p < 0.01, ***p < 0.001, ****p < 0.0001, n = 6 per group **(G–K)** The mRNA levels of NLRP3, GFAP, C3, IL-18 and S100A10. Graphical data are presented as the mean ± S.E.M and significance was determined by one-way ANOVA followed by Tukey’s test. *p < 0.05, **p < 0.01, ***p < 0.001, ****p < 0.0001, n = 6 per group.

After seven consecutive days of morphine injection, the Western blot analyses revealed that repeated morphine treatment elevated the mRNA and protein levels of NLRP3, GFAP and IL-18. Simultaneously, the mRNA and protein levels of C3, a marker for A1 astrocytes, were also heightened, whereas S100A10, a marker for A2 astrocytes, exhibited a notable decrease. However, in comparison to the morphine treatment group, co-administration of MCC950 not only mitigated the increase in protein levels of NLRP3, C3, GFAP, and IL-18 but also reversed the decline in protein levels of S100A10 (Figures 2A–F, one-way ANOVA with Tukey’s multiple comparisons test, n = 6 per group). Additionally, the administration of MCC950 counteracted the alterations in mRNA expression of NLRP3, C3, and S100A10 (Figures 2A, G–K, one-way ANOVA with Tukey’s multiple comparisons test, n = 6 per group).

### 3. Chronic morphine-induced C3 was mainly expressed in astrocyte and MCC950 inhibited the fluorescence expression level of C3 and GFAP

**FIGURE 3:**
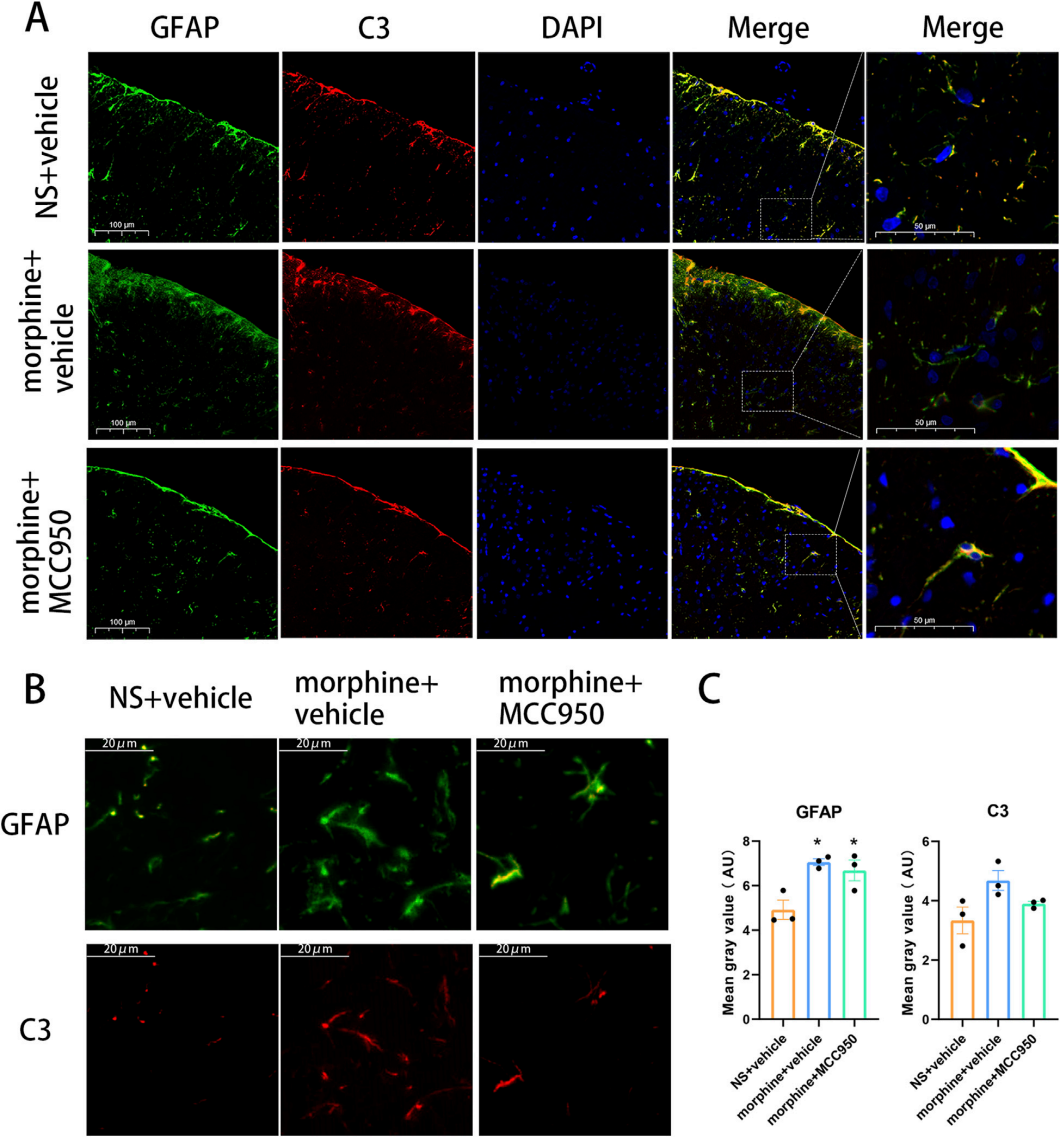
Co-localization of C3 and GFAP in the spinal cord after chronic treatment with morphine. **(A, B)** Representative immunofluorescent images of C3 (red) and GFAP (green) in the dorsal horn of spinal cord of rats treated with saline, morphine and MCC950. **(A)**, Scar bar = 100 µm. **(B)**, Scar bar = 20 µm **(C)** Mean gray value of GFAP and C3, one-way ANOVA followed by Tukey’s test, compared with NS + vehicle group, *p < 0.05, n = 3 per group.

To further assess the impact of reactive astrocytes in morphine-tolerant rats and determine whether the elevated levels of C3 in the spinal cord originate from astrocytes, we conducted immunofluorescence staining to examine the co-localization of C3 and GFAP. The data revealed that C3-positive cells were specifically double-labeled with the astrocyte marker GFAP, confirming that intrathecal administration of morphine activated C3-positive astrocytes. This evidence supports the transformation of reactive astrocytes into A1 astrocytes in morphine-tolerant rats. Additionally, the administration of MCC950 markedly reduced the fluorescence intensity of both C3 and GFAP, suggesting that inhibiting NLRP3 could prevent reactive astrocytes from transforming into the A1 phenotype (Figures 3A, B). Quantification of GFAP and C3 fluorescence intensity showed that seven consecutive days of morphine treatment increased their expression (Figure 3C, one-way ANOVA with Tukey’s multiple comparisons test, n = 3 per group).

## Discussion

To our knowledge, our current study is the first to illustrate the impact of increased NLRP3 expression in the spinal cord on the differentiation of A1/A2 astrocytes in an animal model of morphine tolerance. Furthermore, we illustrated that inhibiting the NLRP3 inflammasome could mitigate the progression of morphine tolerance and revert the differentiation of A1/A2 astrocytes. These findings provide strong evidence supporting the crucial role of the NLRP3 inflammasome in the development of morphine tolerance.

Morphine is a frequently prescribed opioid analgesic in clinical practice. However, long-term use of morphine inevitably leads to analgesic tolerance and hyperalgesia (Fields, 2011). There is an urgent need to discover methods to mitigate the side effects associated with morphine and other related opioids. The mechanism of opioid tolerance is intricate and multifaceted, and there is a growing focus on the role of the neuroinflammatory immune mechanism (Liu et al., 2020).

In our current investigation, we elucidated the role of the NLRP3 inflammasome in orchestrating the differentiation of A1/A2 astrocytes in an animal model of morphine tolerance. The NLRP3 inflammasome, recognized as a multimeric protein complex, mediates the maturation and secretion of interleukin (IL)-1β and IL-18 (Sharma and Kanneganti, 2021). Simultaneously, a mounting body of evidence suggests the involvement of the NLRP3 inflammasome in a broad spectrum of inflammatory diseases. It is noteworthy that numerous studies have underscored the significance of IL-1β as a crucial signaling molecule in the development of morphine tolerance, emphasizing that targeting IL-1β holds potential therapeutic benefits in mitigating morphine tolerance (Johnston et al., 2004; Shavit et al., 2005). Given IL-1β′s pivotal role in the development and progression of morphine tolerance, the NLRP3 inflammasome emerges as a noteworthy target for prospective interventions in the treatment of morphine tolerance. Studies have demonstrated that microglial Toll-like receptor 4 (TLR4) and P2X7 receptor (P2X7R) play a crucial role in the development of morphine tolerance by modulating NLRP3 inflammasome (Wang et al., 2020). Furthermore, research has indicated that NLRP3 knockout mice display resistance to morphine-induced analgesic tolerance, suggesting that morphine may induce tolerance through the Toll4-TAK1-NLRP3 pathway (Wang et al., 2021). Additionally, it has been observed that procyanidins can mitigate morphine tolerance by inhibiting the activation of the NLRP3 inflammasome (Cai et al., 2016). Consistent with previous studies, our experiment involved inducing an animal model of morphine tolerance through intrathecal injection, revealing an upregulation of NLRP3 expression in the rat spinal cord. This finding affirms the involvement of NLRP3 activation in the development of morphine tolerance.

Glia cells such as microglia and astrocytes, can undergo activation due to the prolonged exposure to opioid drugs. Furthermore, the administration of corresponding inhibitors not only diminishes the activation of these glial cell types but also mitigates the development of opioid tolerance (Song and Zhao, 2001). In this experiment, we induced opioid tolerance in rats through continuous intrathecal injections of morphine over a 7-day period. We observed an increase in the expression of the common marker of astrocyte proliferation, glial fibrillary acidic protein (GFAP), confirming the activation of astrocytes during the development of morphine tolerance. Simultaneously, we noted an elevation in the expression of the A1 astrocyte marker, C3. It is noteworthy that although we observed an increase in C3 at both the protein and mRNA levels, no statistically significant differences were found in the fluorescence intensity of C3. This discrepancy may be due to the limited sample size, as the primary goal of this experiment was to observe GFAP and C3 co-localization. With only three samples per group, the statistical power may not have been sufficient to detect significant changes in C3 fluorescence intensity. However, considering the consistency between the immunofluorescence and Western blot results, we believe that increasing the sample size could reveal a statistically significant difference in C3 intensity. Previous studies in other models have shown that the NLRP3 inflammasome has the capacity to induce astrocyte transformation into an A1 phenotype via IL-18 (Hou et al., 2020). The modulation of the NLRP3 inflammasome has been shown to diminish the production of A1 astrocytes triggered by LPS (Darvish et al., 2022). In our experiment, we observed an increase in IL-18 and C3 expression in the morphine group of rats, and increased IL-18 may elucidate the increase in A1 astrocytes induced by the NLRP3 inflammasome. In a model involving chronic intermittent hypoxia, the NLRP3 inflammasome exhibited regulatory control over astrocyte activation, steering it towards an A1 phenotype. Administration of the NLRP3-specific inhibitor, MCC950, demonstrated a reduction in A1 astrocytes and a concurrent increase in A2 astrocytes (She et al., 2022). To the best of our knowledge, this is the first study to confirm that A1 astrocyte phenotype also participated in the development of morphine tolerance. Our observations showed that intrathecal administration of MCC950 resulted in a decline in C3 expression and a reversal in S100A10 expression, suggesting that the NLRP3 inflammasome has the potential to impede reactive astrocytes from transforming into A1-type astrocytes. Consequently, the activation of A1 astrocytes may serve as a promising therapeutic strategy for addressing morphine tolerance.

In our study, the observation that morphine-induced antinociceptive tolerance could be alleviated by co-administration of MCC950 has provided the basis for considering MCC950 as a potential suppressor of morphine antinociceptive tolerance in clinical trials. Meanwhile, the increase in A1 astrocyte marker suggest that the activation of reactive astrocytes could be a target for counteracting morphine tolerance. Our study has several limitations that should be acknowledged. First, although selecting a single time point (day 7) for assessing mRNA and protein levels is consistent with established protocols, it may not fully capture the temporal dynamics of molecular changes. These changes could occur at earlier stages. For instance, additional time points, such as days 3 and 5, might have provided a more detailed understanding of the progression of morphine tolerance and the associated molecular alterations. Another limitation of our current study is the lack of investigation into the specific mechanism through which NLRP3 promotes the differentiation of A1 astrocytes. Previous research has indicated that IL-18 can induce the transformation of astrocytes into the A1 phenotype via the NF-κB pathway (Hou et al., 2020). In our experiment, the increase of IL-18 in spinal cord may provide insight into the mechanism by which NLRP3 promotes the differentiation of reactive astrocytes. However, whether other pathways might also be involved in this process remains to be further studied. Meanwhile, we only explored the co-labeling of the A1 astrocyte marker C3 with GFAP. Including the co-labeling of S100A10 with GFAP would provide a more comprehensive result. This may guide future research.

In summary, our findings indicate that chronic morphine treatment induces substantial analgesic tolerance and activates the NLRP3 inflammasome. The adverse effects of morphine can be mitigated by the NLRP3 inhibitor MCC950, which also hinders the development of morphine-induced analgesic tolerance. Our results offer a potential novel solution to alleviate analgesic tolerance, highlighting A1 reactive astrocytes as a promising therapeutic strategy for addressing morphine tolerance.

## Data Availability

The original contributions presented in the study are included in the article/supplementary material, further inquiries can be directed to the corresponding authors.

## Abbreviations

NLRP3, nucleotide-binding oligomerization domain-like receptor pyrin domain-containing-3; ASC, apoptosis-associated speck-like protein containing a caspase-recruitment domain; TLR4, Toll-like receptor 4; GFAP, glial fibrillary acidic protein; AD, Alzheimer’s disease; PD, Parkinson’s disease; ALS, Amyotrophic lateral sclerosis.
